# Inflammatory myofibroblastic tumor of epididymis: a case report and review of literature

**DOI:** 10.1186/1477-7819-6-119

**Published:** 2008-11-11

**Authors:** Pankaj P Dangle, Wenle Paul Wang, Kamal S Pohar

**Affiliations:** 1 The James Cancer Hospital and Solove Research Institute, Ohio State University and Comprehensive Cancer Center, Columbus Ohio, 43210, USA; 2 Department of Pathology, The Ohio State University, Columbus Ohio, 43210, USA; 3 Department of Urology, The James Cancer Hospital and Solove Research Institute, Ohio State University and Comprehensive Cancer Center, Columbus Ohio, 43210, USA

## Abstract

**Background:**

Epididymal inflammatory myofibroblastic tumor, also known by various other synonyms is a rare benign disease. Only eight cases have been reported to date. The most common presentation is a scrotal mass of variable duration. For a scrotal mass it is difficult to distinguish a benign or malignant etiology, in addition to the origin whether from testis or epididymis. As a result the definitive diagnosis can only be established by surgical exploration.

**Case presentation:**

We report the ninth case of epididymal IMT who based on clinical and radiological findings underwent radical orchidectomy, with the histology suggestive of inflammatory myofibroblastic tumor. At 4 years follow up the patient is free of disease recurrence.

**Conclusion:**

IMT though rare should be considered in the differential diagnosis of epididymal mass. Clinically it is often difficult to distinguish the origin of mass and even though the disease has benign nature and course it is crucial to counsel patients for orchidectomy as definitive diagnosis is established on surgical exploration.

## Background

Inflammatory Myofibroblastic tumor (IMT) of epididymis is a distinct but rare entity. IMT is also described by other synonyms, more commonly as inflammatory pseudotumor. Extra genitourinary and genitourinary sites are well documented with various proposed etiological theories [1]. Epididymal IMT is rare and only eight cases are reported in the literature [2-8]. The most common reported presentation of epididymal IMT is lump in the scrotum. Due to its uncertain etiology many of these patients have been offered antibiotics with no clinical response. We describe a case of a young healthy male with a painless indurated scrotal mass with possible involvement of the testicle. Based on patient's age and clinical findings the lump was suspected to be a testicular tumor and therefore was subjected to radical orchidectomy. We present our case and review of literature for epididymal IMT.

## Case presentation

A 22 year old healthy Caucasian male noticed a swelling and a palpable mass in the right scrotum for a period of one week. Patient denied any history of fever, trauma, urethral discharge and any previous history of recurrent urinary tract or sexually transmitted infections. There was no past history of exposure to tuberculosis. Physical examination revealed a nontender indurated solid mass in the lower pole of right testicle possibly also involving the epididymis.

Scrotal ultrasound demonstrated a solid heterogeneous mass involving right testicle with possible extratesticular extension into the epididymis. Quantitative serum Beta -human chorionic gonadotropin, alpha-fetoprotein and LDH (lactate dehydrogenase) were within normal limits.

**Figure 1:**
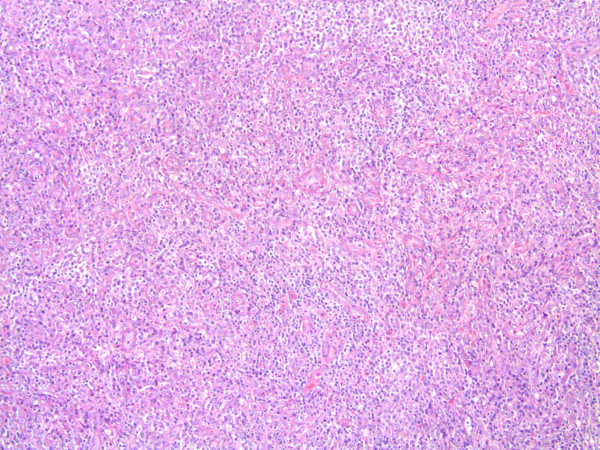
**Low magnification of IMT (100×).** Spindle cells mixed with inflammatory cells. The spindle cells are epithelioid, mixed with chronic inflammatory cells. The myoepithelial cells are loosely arranged. There is increased vascularity in the IMT.

**Figure 2:**
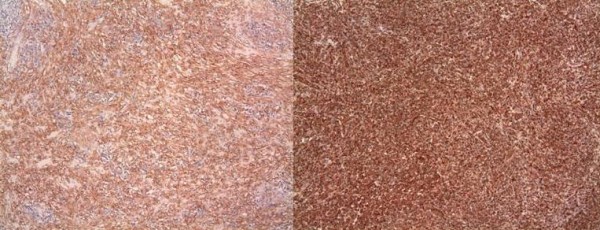
(Immunostains) – Immunostaining showing spindle myoepithelial cells positive for smooth muscle actin and vimentin.

**Figure 3:**
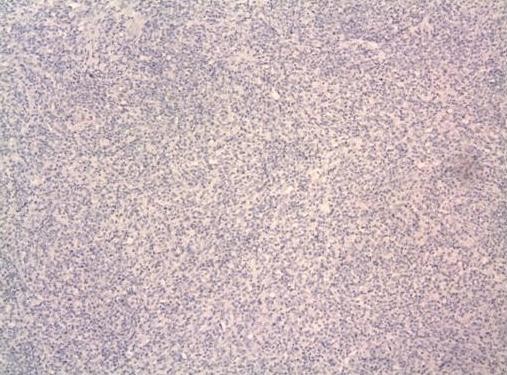
(Immunostain) – Immunostaining negative for ALK-1.

With the presumed diagnosis of testicular tumor a right radical orchidectomy was performed. On gross pathologic examination the mass was abutting the tunica albugenia but further examination revealed being confined to epididymis with normal testicular parenchyma. Histology of the mass (4 × 2.2 × 1.8 cm) demonstrated a spindle myoepithelial and polygonal cell proliferation with intense lymphoplasmacytic infiltrate. (Fig. 1) The mass also revealed scattered neutrophils with positive immunostaining for smooth muscle actin, vimentin (Fig. 2), CD3, CD20, CD68 and AE1/AE3 but was negative for ALK-1 (Fig. 3) and CD 138. There was presence of numerous T cells, B cells and macrophages but absence of atypical epithelial cells. The lesion also lacked presence of sperm, Michaelis Gutman bodies, GMS (Grocott's silver) and AFB (acid fast bacilli) stain for any fungal or acid fast organism respectively. The histological and staining pattern was consistent with inflammatory myofibroblastic tumor of the epididymis. The reagents, their source, pre-treatment, dilution and incubation times are listed in table 1.

**Table 1:** Immunohistochemical reagents used in our case.

**Antibody specificity**	**Vendor/Source**	**Cat. Number/Clone**	**Pretreatment**	**Dilution**	**Incubation time(minutes)**
CD3	Dako	A0452/Rabbit	TRS pH6/30 sec in Pressure cooker	1 in 400	30

CD20	Dako	M0755/L26	TRS pH6/30 sec in Pressure cooker	1 in 200	30

CD68	Dako	M0814/KP1	TRS pH6/30 sec in Pressure cooker	1 in 3000	30

SMA	Dako	M0851/1A4	No	1 in 400	30

ALK-1	Dako	M7195/ALK-1	TRS pH6/30 sec in Pressure cooker	1 in 50	30

CD138	Dako	M7228/MI15	TRS pH6/25 min in steamer	1 in 90	30

Vimentin	Dako	M0725/V9	TRS pH6/25 min in steamer	1 in 200	30

The patient recovered well with no evidence of any recurrence at the site of resection or other sites after 4 years of follow-up.

Based on such an unusual and rare finding a thorough Medline search revealed eight additional patients with similar presentation of scrotal lump. All patients had exploration of the scrotal mass due to its solid heterogenous features on ultrasound and clinical examination. All patients underwent either excision of mass or radical orchidectomy.

## Discussion

Inflammatory Myofibroblastic tumor (IMT) is a well described disease and can occur in many organs such as lung, skin, soft tissues, breast, gastrointestinal tract, pancreas, oral cavity, bone and central nervous system. However various sites in genitourinary tract have also been reported but less commonly [1], epididymis is least common with only 8 cases (20 to 73 years) being reported to date [2-8].

Only those tumors with spindle myoepithelial cell proliferation and lymphocytic infiltrate qualify for IMT. Various synonyms like inflammatory pseudo tumor, plasma cell granuloma, plasma cell pseudo tumor, atypical Myofibroblastic tumor and post operative spindle nodule are used interchangeably [1]. In spite of this the term inflammatory myofibroblastic tumor is preferred as inflammatory pseudo tumor has been applied to diverse entities like reparative pseudosarcomatous lesion of lower genitourinary tract [9], infectious etiology like mycobacterium avium intracellulare and Epstein Barr virus (EBV) [10,11]. The post operative spindle cell nodule [1] denotes to spindle cell proliferation with easily identifiable mitotic figures deposited in a less conspicuous myxoid background where as a classic IMT describes a lesion characterized by spindle cell proliferation in a loose, edematous myxoid stroma associated with granulation tissue type and a mixed acute and chronic inflammatory cells composed of lymphocytes, plasma cells, eosinophils with occasional neutrophils and mast cells.

The pathophysiology of IMT is not well understood, various etiologies have been proposed including a reparative process related to delayed chronic response to remote or undetected trauma [1]. Infectious etiologies such as Epstein Barr virus, mycobacterium avium intracellulare and herpes virus 8 have also been suggested to be associated as an etiological agent with IMT [10-12]. However no such similar association of EBV as an etiological agent has been demonstrated with epididymal IMT [4]. Cytogenetic studies show that some IMT (mediastinal and abdominal) lesions have genetic clonal abnormality at chromosome region 2p22–24 with breakage in band p22–24, with specific involvement of 2p23, suggesting a neoplastic change [13]. In some of the IMTs an anaplastic lymphoma kinase (ALK) gene on 2p23 has been implicated in pathogenesis of this lesion. A fluorescence in situ hybridization with a probe flanking the ALK gene at 2p23 demonstrated translocation of ALK gene. An immunohistochemical staining for ALK showed positive cytoplasmic staining in the myofibroblastic cells [13,14]. Two case reports [7,8] including ours have studied ALK immunostaining on epididymal tissue with none staining positive for ALK.

Patients with IMT can present with fever, night sweats, weight loss, malaise or abnormal laboratory parameters such as elevated ESR (erythrocyte sedimentation rate), anemia, leukocytosis and site specific symptoms [15]. However patients with IMT of epididymis rarely present with above symptoms but most commonly with a palpable mass of variable duration ranging from 3 weeks to 5 years [2,4]. The mass is clinically often indistinguishable from the testis. One patient described in the literature, clinically had multiple [5] extra testicular masses, with 3 in the body of the epididymis, 1 at head of the epididymis and 1 in tunica vaginalis on subsequent exploration [2]. Our patient presented with 1 week history of a palpable mass with no precedent history of trauma and recurrent urinary or sexually transmitted infections. A summary of all reported eight cases and our case has been presented in table 2. Based on the clinical examination the differential diagnosis of such a mass is testicular tumor, adenomatoid carcinoma, paratesticular sarcoma, epididymal adenocarcinoma.

**Table 2:** Brief summary of cases reported in the literature.

**Reference**	**Age**	**Presentation**	**Immunomarkers**		**Treatment**	**Follow-up**
					
			**Positive**	**Negative**		
Orosz et al [5]	63	Left Scrotal mass	α-smooth muscle actin, muscle specific actin, vimentin, kappa and lambda chain	Desmin, S-100, Factor VIII-related antigen,	Radical Orchidectomy	_

Lam et al [4]	43	Rt Scrotal mass	Vimentin, smooth muscle actin	Desmin, cytokeratin	Initial antibiotic, Surgical excision as definitive treatment	At 6 months follow-up no recurrence

Chan et al [6]	43	Rt. Scrotal mass	Polyclonality of plasma cells for Light chains		Radical Orchidectomy	_

	20	Left Scrotal mass	Polyclonality of plasma cells for Light chains		Excision of lump from tail of epididymis	_

Brauers et al [3]	73	Left Scrotal mass	Vimentin, α1anti-chymotrypsin, CD 68, α-smooth muscle actin	Desmin, myoglobin, myosin	Epididymectomy	_

Cooperman et al [2]	30	Rt. Scrotal mass	_	_	Excision of masses	_

Kapur et al [7]	36	Rt. Scrotal mass and rt. Inguinal lymphadenopathy	Vimentin, smooth muscle actin,	Cytokeratin (AE1/AE3), muscle specific actin, desmin, CD34 ALK, inhibin	Radical Orchidectomy	_

Stylianos et al [8]	45	Left Scrotal mass	Smooth muscle cell specific actin, Desmin	CD34, S-100, cytokeratin, AE1/AE3, ALK	Radical Orchidectomy	No recurrence at 3 year follow-up

Our case	22	Rt. Scrotal mass	Vimentin, smooth muscle actin, CD3, CD20, CD 68, AE1/AE3	ALK-1, CD 138	Radical Orchidectomy	No recurrence at 4 year follow-up

The diagnosis of IMT is based on the histological features of spindle myoepithelial cell proliferation, lymphocytic and inflammatory infiltration. Other immunomarkers could substantiate the diagnosis of IMT. Immunomarkers such as vimentin, actin and CD 68 are positive in 25% cases [1]. A similar finding was noted by Brauers and Lam et al [3,4] in epididymal IMT, with immunostaining being positive for vimentin, actin, CD 68 and α1-anti chymotrypsin. In our patient histology stained positive for vimentin, smooth muscle actin and CD 68 but negative for ALK-1 and CD138.

Various non surgical treatment options have been proposed at sites other than genitourinary tract including cyclosporine, corticosteroid, methotrexate, antibiotics [16-18] and radiation [19] with variable success. Spontaneous regression has also been reported [15]. Surgical excision is definitive to exclude malignant etiology for scrotal masses. Our patient and most patients [4-8] described in the literature had orchidectomy as a final treatment. Though Cooperman et al [2] described local excision of clinically evident extra testicular masses with a normal testicle confirmed on ultrasound, the frozen section of these masses excluded presence of malignancy. Similarly Brauers et al [3] report epididymectomy for a clinically palpable 1 cm mass with normal testis on examination. Lam et al [4] however performed orchidectomy for a firm scrotal mass clinically indistinguishable from testis. In our patient based on clinical examination and ultrasound, it was difficult to justify local excision due to difficulty in differentiating whether the mass was separate from testis.

The abdominal and retroperitoneal variant presents with more aggressive pattern compared to their extra abdominal counterparts, with recurrence rate of 23–37% [15,20]. The true potential for metastasis as reported by Coffin et al [15] in their series of 84 patients is unclear whereas Meis and Enzinger [20] reported cases with metastasis. The reason for such inconsistent finding is uncertain, whether it represents multifocal disease is unclear at present [15,20]. However recurrence of epididymal IMT has not been reported to date. Our patient is free of any recurrent disease at previous site of excision or other distant sites at end of 4 years of follow-up.

## Conclusion

IMT though rare should be considered in the differential diagnosis of epididymal mass. Clinically it is often difficult to distinguish the origin of mass either from testis or epididymis. Radiological studies are unable to differentiate benign or malignant nature and as a result definitive diagnosis is established on surgical exploration. Depending on the gross characteristics and frozen section of clinically distinct masses, either a local excision or radical orchidectomy is offered. Thus even though the disease has benign nature and course it is crucial to counsel patients for orchidectomy as definitive diagnosis is established on surgical exploration.

## Consent

Written informed consent was obtained from the patient for publication of this case report and any accompanying images. A copy of the written consent is available for review by the Editor-in-Chief of this journal.

## Competing interests

The authors declare that they have no competing interests.

## Authors' contributions

PPD was involved in conception and design, acquisition of data, data analysis, and interpretation, manuscript drafting and final approval. WPW was involved in acquisition of data, data analysis, provided pathologic imaging, interpretation of data and final approval. KSP was involved in conception and design, acquisition of data, data analysis, and interpretation, manuscript drafting and final approval.
